# Predictors of circuit health in neonatal patients receiving extracorporeal membrane oxygenation (ECMO)

**DOI:** 10.1038/s41598-022-05389-3

**Published:** 2022-01-24

**Authors:** Rita G. Hazboun, Nada Darwish, Gianna Rotyliano-Sykes, Nayef Chahin, Jie Xu, John Miller, Christos Calaritis, Leroy Thacker, Russell Moores, Karen D. Hendricks-Muñoz

**Affiliations:** 1 Division of Neonatal Medicine, Department of Pediatrics, Children’s Hospital of Richmond at VCU, Virginia Commonwealth University School of Medicine, PO Box 980646, Richmond, VA 23298-0646 USA; 2 Department of Pediatrics, Children’s Hospital of Richmond at VCU, Virginia Commonwealth University School of Medicine, Richmond, VA USA; 3 Virginia Commonwealth University School of Medicine, Richmond, VA 23298 USA; 4 Pediatric Cardiology, Children’s Hospital of Richmond at Virginia Commonwealth University Health System, Richmond, VA 23219 USA

**Keywords:** Cardiology, Risk factors

## Abstract

To identify predictors of neonatal ECMO circuit health, a retrospective analysis of circuit functional pressure and flow parameters as well as infant clotting values were collected 48 h prior to and 24 h post circuit change. Circuit impairment was defined as need for partial or total circuit change. Statistical analysis used multivariate statistics and non-parametric Mann–Whitney U-test with possible non-normality of measurements. A total of 9764 ECMO circuit and clotting values in 21 circuits were analyzed. Circuit delta-P mean, and maximum values increased from 8.62 to 48.59 mmHg (p < 0.011) and 16.00 to 53.00 mmHg (p < 0.0128) respectively prior to need for circuit change. Maximum and mean Pump Flow Revolutions per minute (RPM) increased by 75% (p < 0.0043) and 81% (p < 0.0057), respectively. Mean plasma free hemoglobin (pfHb) increased from 26.45 to 76.00 mg/dl, (p < 0.0209). Sweep, venous pressure, and clotting parameters were unaffected. ECMO circuit delta-P, RPM, and pfHb were early predictors of circuit impairment.

## Introduction

Extracorporeal membrane oxygenation (ECMO) is a lifesaving procedure improving survival in neonates and pediatric patients with cardiorespiratory failure that is refractory to conventional therapy^1–3^. Leading causes of patient morbidity and mortality include hematological complications of bleeding and thrombosis associated with ECMO circuit dysfunction^4,5^. Clot formation within the ECMO circuit is the most common mechanical complication, seen in greater proportion of neonatal patients and affecting as much as 38–50% of neonatal and 28–37% of pediatric patients on ECMO^6^. Furthermore, membrane oxygenator clot formation and subsequent oxygenator failure occurs with a higher incidence in pediatric patients, 1 in 5, as compared to adults with less than 1 in 10 occurrences^7^. Patients on ECMO have a higher predisposition for clot formation due to blood stream activation of the clotting cascade, triggered by foreign surface contact within the ECMO circuit tubing, in conjunction with the patient’s underlying illness^8,9^. Clot formation can result in oxygenator or pump obstruction leading to circuit failure. As the neonate on ECMO is completely dependent on circuit function for life support, malfunction or circuit inefficiency can have catastrophic consequences^4,5^.

Unanticipated changes in the ECMO circuit components can place the patient at risk for hemodynamic instability and hypoxic end-organ damage^10^. These risks are more likely to occur if the circuit change is needed without ample warning as an emergency measure^11^. Thus, the ability of healthcare providers to identify early clot formation and need for circuit component or complete circuit replacement prior to obstruction is crucial.

Early identification of ECMO circuit health markers can potentially assist in prediction of risk or need for complete or partial circuit change. Previous evaluations of possible circuit health indicators identified significant associations of increased shunt flow with distal circuit obstruction^7^, clotting parameters of increased d-dimer^10,11^, decreased fibrinogen, decreased platelet count, decreased heparin dose, as well as increased circuit sweep with subsequent need for oxygenator change^10^. All studies were conducted on adult and pediatric populations who differ from neonatal patients in terms of diagnosis, coagulation physiology and clotting risks^10,12^.

In this study, we aimed to determine markers associated with circuit health dysfunction or with continued circuit optimal performance within the neonatal population. We hypothesized that increased delta-P, RPM, pfHb and decreased circuit flow, platelet count and fibrinogen are reflective of circuit health and can assist in early prediction of circuit health or circuit failure risk in the neonatal patient. Key areas of determination were timing of occurrence of identifiable circuit parameters and individual coagulation values associated with impending need for circuit change in this high risk ECMO population.

## Methods

A retrospective review of medical records of infants who received ECMO support from January 1, 2015 to December 31, 2019 at the Children’s Hospital of Richmond at Virginia Commonwealth University (CHoR at VCU) level IV NICU was conducted. The study received approval by the VCU Institutional Review Board.

All methods were carried out in accordance with guidelines and regulations.

Exclusion criteria included infants whose care did not include collection of circuit or coagulation parameters. During the years of analysis, critical parameters including delta-P were not documented. Patients without detailed parameters were excluded from analysis. Critical parameters including delta-P became standard documentation in 2017. Data collected included: demographic, clinical, ECMO circuit and laboratory data. Demographic variables obtained included: date of birth, race, gestational age at birth, birth weight, and gender. Clinical parameters included: diagnosis that led to ECMO, ECMO type [Veno-Venous (VV) vs Veno-Arterial (VA)], number of days on ECMO, identification of clots on any day, circuit changes, heparin dose, and survival. Clotting parameters collected included hemoglobin, platelet count, fibrinogen, activated clotting time (ACT), partial thromboplastin time (PTT), prothrombin (PT), anti Xa, anti-thrombin III, international normalized ratio (INR) and plasma free Hb (pfHb)**.** Infants were divided into those who received a circuit-change and those who never required a circuit change. Infants who never required a circuit change were considered the control arm. Infants who required a partial or complete ECMO circuit change were considered in the circuit-change arm. Circuit changes were identified by review of the medical chart of each ECMO patient. Each circuit change was treated as a separate event. According to VCU Institutional Review Board, due to the nature of the study informed consent was not required. Data analyzed included patient demographic characteristics, type and duration of ECMO performed and clinical diagnoses, clinical outcomes, laboratory coagulation values, and ECMO circuit specific parameters. ECMO circuit values were identified every 15 min for a total of 4 values per hour analyzed 48 h prior to the ECMO circuit change and through the 24 h post circuit change. In those infants without circuit change circuit values every 15 min were identified throughout the circuit run duration with the lowest and highest value recorded throughout the ECMO circuit run.

Coagulation parameters performed during circuit runs were analyzed with specific notation of values at 48 h, 24 h, 12 h, 6 h prior to circuit change (timeframe was selected randomly), and hourly post circuit change through the 24 h after circuit change (based on our unit practice). In those without circuit change all levels were analyzed with identification of minimum, maximum and mean.

The standard practice in our NICU is to obtain a complete blood count and anti-Xa level every four hours, anti Xa level after 2 h from Heparin dose change and to obtain coagulation parameters, pfHb and antithrombin-III activity % every 12 h. ACT was measured hourly. Anti-Thrombin III was administered, and transfusions of packed red blood cells, platelets, and cryoprecipitate were given as needed and per the institutional protocol. Current NICU practice is to transfuse for Hemoglobin level less than 10.0 g/dL, and platelets less than 100 × 10^9^/L.

The change in laboratory parameters, the maximum and minimum measured over the 48 h prior to ECMO circuit change were evaluated—against control subjects and against the parameter values immediately and 24 h post circuit change, as a potential predictor for the need for a circuit change.

### ECMO circuit and monitoring

Standard ECMO practice includes infants treated with either Veno-Arterial (VA) or Veno-Venus (VV) ECMO using a heparin-coated circuit, with Quadrox oxygenator and Rotaflow centrifugal blood pump (both Maquet Cardiopulmonary AG, Germany). Standard management included systemic anticoagulation administered with a target ACT of 160–220 s, a target anti-Xa level of 0.3–0.7 IU/mL, and a target antithrombin-III activity of 50–80% (≤ 30 days old) and 80–120% (> 30 days old) per our institution’s guidelines. Anticoagulation targets were tailored to each infant throughout the course of ECMO.

### Statistical analysis

Data was analyzed with the Statistical Analysis System (SAS). Data analysis included descriptive statistics with mean and standard deviation (SD) for numerical variables, and percentage within different categories for categorical variables. Group comparisons were applied using the Chi-squared (χ^2^) test or Fisher's exact test for categorical variables and ANOVA for continuous variables. Laboratory parameters were evaluated using non-parametric Mann–Whitney U-test with possible non-normality of measurements. Each circuit change was regarded as a separate event, even when occurring for the same patient.

## Results

### Study population

A total of 53 infants were identified as having ECMO care during the study period with 32 infants excluded due to lack of documentation of ECMO circuit parameters yielding a total of 21 infants included in the study. Of these 21 infants a total of 39 circuit analyses were performed. In the circuit change group, 25 circuit change events occurred in 7 infants. 288 data point values were identified per circuit change. Total values of circuit data points analyzed was 7200. In the 14 infants in the no-circuit change group a total of 2064 data point values were used for analysis for a total of 9764 circuit values.

### Patient demographics and circuit characteristics

There was no statistically significant difference between the circuit change or no-circuit change groups related to gestational age, birth weight or race (Table 1). There was no statistically significant difference between the two groups regarding the type of ECMO (VV or VA) received. Diagnosis leading to ECMO (meconium aspiration, pulmonary hypertension, left and right congenital diaphragmatic hernias, and congenital heart disease) did not differ between groups and were not associated with any statistically significant risk of circuit change (Table 2). Infants in the circuit change group were more likely to have longer duration of ECMO care compared to those in the no-circuit change group, with 12.7 ± 4.7 days for the circuit change group and 6.0 ± 3.0 days for the control group, p < 0.01.

**Table 1:** Patients’ demographics (n = 21).

	Patients with circuit change (n = 7)	Patients without circuit change (n = 14)
Birth gestational age^a^ (weeks ± SD)	35.9 ± 4.98	36.8 ± 4.29
Birth weight (g ± SD)	2258 ± 1319	2880 ± 1022
**Sex**		
Male, n (%)	5 (71)	6 (55)
**Race**		
African American, n (%)	5 (71)	6 (55)
White, n (%)	1 (14)	2 (18)
Hispanic, n (%)	0	1 (9)
Other, n (%)	1 (14)	2 (18)

^a^Premature infants were placed on ECMO weeks–months after birth.

*SD* Standard Deviation.

**Table 2:** Patients’ diagnoses (n = 21).

Diagnosis	Patients with circuit change (n = 7)	Patients without circuit change (n = 14)
Meconium aspiration	4	3
Left congenital diaphragmatic hernia	1	2
Right congenital diaphragmatic hernia	0	2
Pulmonary hypertension	2	5
Congenital heart disease	0	2

There was no statistically significant risk related to diagnosis and circuit change risk.

### ECMO circuit parameters

**Figure 1:**
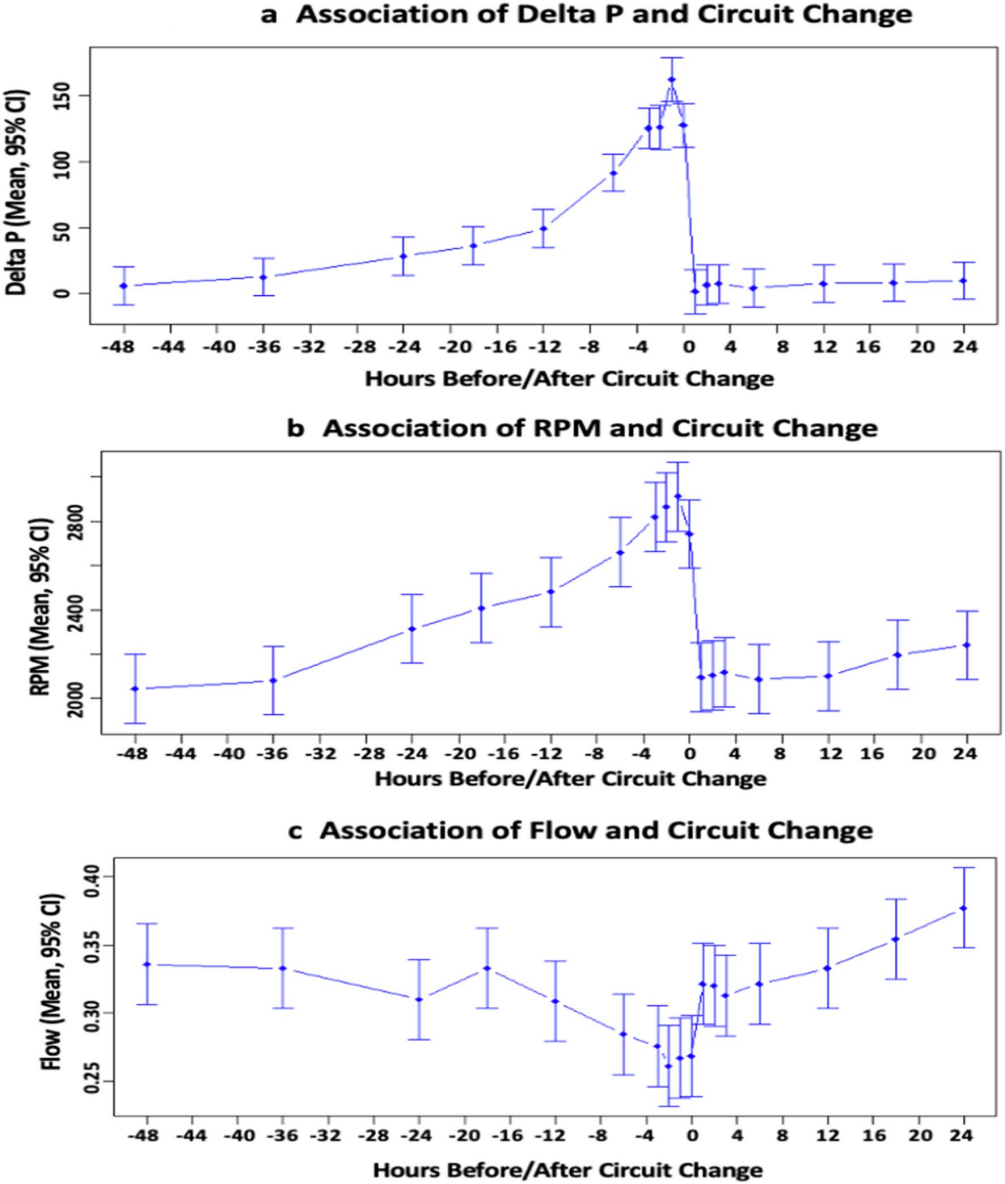
Associations of circuit parameters with circuit change. ECMO circuit mean and SD parameter changes over time before impairment (change) of circuit at 0 h of time and 24 h post circuit change. (**a**) Circuit Delta-P mean and SD values over time (p < 0.011 and < 0.012 respectively), (**b**) Circuit RPM mean and SD values (p < 0.004 and p < 0.005 respectively), (**c**) Circuit Flow mean and SD values (p < 0.0001). *RPM* Revolutions per minute, *SD* Standard deviation.

In the evaluation of circuit pump parameters several areas were associated with statistically significant differences in the two groups (Table 3). Overall, as shown in Fig. 1, the mean and maximum delta-P were significantly increased in the circuit- change group compared to the no-circuit change group, p < 0.011 and < 0.012, respectively (Fig. 1a). The maximum RPM and the mean RPM were increased in the circuit-change group compared to the no-circuit change group, p < 0.004 and p < 0.005, respectively (Fig. 1b). ECMO circuit Flow significantly decreased prior to need for circuit change in the circuit-change group compared to the no-circuit change group p < 0.0001 (Fig. 1c). ECMO circuit parameters of sweep and venous pressure were not statistically significant in the circuit-change group compared to the no-circuit change group, p values 0.86 and 0.93, respectively.

**Table 3:** Circuit parameter comparisons between circuit change and no-circuit change values. n = 9264 data time points.

Parameter	Circuit change (n = 7200)		Control group (n = 2064)		*p*-value
	Median	[Min, Max]	Median	[Min, Max]	
Delta P min (mmHg)	0.00	[− 7.00, 6.00]	0.00	[− 16.00, 1452.00]	0.8918
Delta P max	153.00	[12.00, 197.00]	16.00	[− 12.00, 45.00]	0.0110
Delta P mean	48.59	[6.71, 63.29]	8.62	[− 4.30, 32.70]	0.0128
RPM min	1830.00	[1725.00, 2160.00]	1780.00	[1235.00, 2095.00]	0.2766
RPM max	3000.00	[2170.00, 3395.00]	2270.00	[1970.00, 2611.00]	0.0043
RPM mean	2452.00	[1965.00, 2667.50]	1987.50	[1827.50, 2285.00]	0.0057

*RPM* revolutions per minute, *Max* maximum, *Min* minimum.

There was a strong association of elevated delta-P compared to individual baseline and subsequent need for circuit change. This maximum delta-P increase above baseline occurred within 6 h prior to the need for circuit change with a return to individual baseline values after the circuit change, p < 0.01.

### Hematologic and clotting parameters

The mean pfHb was significantly increased in the circuit-change group compared to the no-circuit change group, prior to need for circuit change p < 0.02 (Table 4). However, there was no statistically significant increase in the maximum pfHb in the circuit-change group compared to the control group. There were no differences in need for heparin dose changes or dose between the two groups. The mean, maximal or minimum levels of ACT, hemoglobin values, platelet count, fibrinogen level, PT, PTT, INR, Antithrombin-III activity % and anti Xa levels did not differ between the circuit change or no-circuit change group**.**

**Table 4:** Clotting parameter comparisons between circuit change and no-circuit change values. n = 9264 data time points.

Parameter	Circuit change (n = 7200)		Control group (n = 2064)		*p*-value
	Median	[Min, Max]	Median	[Min, Max]	
Plasma free Hb min	22.00	[11.90, 28.00]	11.90	[11.90, 116.00]	0.0842
Plasma free Hb max	137.00	[34.90, 420.00]	41.00	[11.90, 277.00]	0.0569
Plasma free Hb mean	76.00	[28.00, 224.00]	26.45	[11.90, 196.50]	0.0209

*Hb* Hemoglobin, *Max* maximum, *Min* minimum.

## Discussion

Despite its critical need, ECMO circuit failure, including the need to change the oxygenator, the pump, or the entire ECMO circuit continues to be a persistent challenge associated with increased risk for morbidity and mortality^11^. As such, identification of markers of risk remain crucial to assist providers in critically assessing circuit malfunction. Our study identifies several parameters that are elevated in association with ECMO circuit dysfunction and need for circuit change. To our knowledge this is the first study to identify early circuit functional parameter changes in routinely collected circuit delta P, RPM and Flow associated with prior need for circuit change in the neonatal population. Furthermore, average pfHb levels were also notably elevated prior to the need for circuit change. In this neonatal population, our results did not detect previously identified changes in any other clotting factors that had been associated with adult and pediatric circuit change studies or detection of D dimer, platelets count, and fibrinogen associated with circuit health^10,11^. This difference is likely due to our population coagulation differences as well as potentially to our current therapeutic guidelines in care management that address alterations in many coagulation values in the neonate which may differ as compared to adults or pediatric patients^5,10,12^.

Currently, no reliable method exists for assessing neonatal ECMO circuit health or predicting optimal ECMO circuit efficiency or impending circuit malfunction. As thrombosis is a major concern for circuit health^4,5^, providers rely on visual inspection for thrombosis within areas of the circuit to detect potential problems such as a clot in the oxygenator^13^. This visual inspection, though necessary is prone to human error, difficult to measure, quantify or use for prediction of circuit health. Given the high ECMO circuit malfunction risk that the neonate on ECMO experiences^6,7^, finding objective markers of ECMO health could be instrumental in avoiding ECMO dysfunction morbidities. The ECMO circuit parameters studied in these investigations are routinely documented in every neonatal patient on ECMO. Despite this routine documentation, these parameters are likely not identified and trended as potential markers of circuit health. ELSO recommendations indicate that patient specific clotting parameters should be trended for every neonatal patient on ECMO, and adjustments made accordingly^14^. Based on our study, additional trending of baseline circuit flow, delta P and RPM potentially can contribute to early identification of circuit health in the neonatal population. Our study has an inherent weakness due to the nature of its retrospective design and the small sample size. However, all circuits were evaluated including those without need for circuit change on a minute-to-minute basis to eliminate bias. Additionally, the large dataset of circuit values and the striking and consistent identification of increased circuit parameter changes in those with need for circuit changes remained consistently strong throughout the analysis.

Early indicators of circuit malfunction can be a valuable tool in the neonatologist’s armamentarium with the potential to create an alert to troubleshoot circuit malfunction, arrange an intervention in a timely manner and avoid an urgent rush to intervene in circuit malfunction.

These results provide an opportunity to independently or in conjunction with current clot inspection methodology to bring us closer to identification of circuit health. Prospective studies are needed to validate the utilization of these identified parameters and investigate their usefulness in predicting circuit health. Given the increased risk of ECMO circuit health in this population, our study provides opportunity to assess the health of the ECMO circuit through objective utilization of circuit functional parameters that have not been previously identified to for possible integration in quality and safety management plans for this population.
