# Publisher Correction: Predictors of circuit health in neonatal patients receiving extracorporeal membrane oxygenation (ECMO)

**DOI:** 10.1038/s41598-022-06844-x

**Published:** 2022-02-14

**Authors:** Rita G. Hazboun, Nada Darwish, Gianna Rotyliano-Sykes, Nayef Chahin, Jie Xu, John Miller, Christos Calaritis, Leroy Thacker, Russell Moores, Karen D. Hendricks-Muñoz

**Affiliations:** 1 Division of Neonatal Medicine, Department of Pediatrics, Children’s Hospital of Richmond at VCU, Virginia Commonwealth University School of Medicine, PO Box 980646, Richmond, VA 23298-0646 USA; 2 Department of Pediatrics, Children’s Hospital of Richmond at VCU, Virginia Commonwealth University School of Medicine, Richmond, VA USA; 3 Virginia Commonwealth University School of Medicine, Richmond, VA 23298 USA; 4 Pediatric Cardiology, Children’s Hospital of Richmond at Virginia Commonwealth University Health System, Richmond, VA 23219 USA

Correction to: *Scientific Reports* 10.1038/s41598-022-05389-3, published online 24 January 2022

The original version of this Article contained an error in the author list. Rita G. Hazboun was incorrectly listed twice. The second time the author’s name was listed in error.

The original Article has been corrected.

